# Topical Administration of Pirfenidone Increases Healing of Chronic Diabetic Foot Ulcers: A Randomized Crossover Study

**DOI:** 10.1155/2016/7340641

**Published:** 2016-07-10

**Authors:** Marcela Janka-Zires, Paloma Almeda-Valdes, Ana Cecilia Uribe-Wiechers, Sonia Citlali Juárez-Comboni, Joel López-Gutiérrez, Jarod Jazek Escobar-Jiménez, Francisco J. Gómez-Pérez

**Affiliations:** ^1^Instituto Nacional de Ciencias Medicas y Nutricion Salvador Zubiran, Endocrinology and Metabolism Department, Vasco de Quiroga 15, Colonia Belisario Domínguez Sección XVI, Tlalpan, 14080 Mexico City, CDMX, Mexico; ^2^Grupo Ángeles, Camino a Santa Teresa 1055, Colonia Héroes de Padierna, Magdalena Contreras, 10700 Mexico City, CDMX, Mexico; ^3^Cell Pharma, Calzada de Las Bombas 128, 04980 Mexico City, CDMX, Mexico

## Abstract

Only 30 percent of chronic diabetic foot ulcers heal after 20 weeks of standard treatment. Pirfenidone is a drug with biological, anti-inflammatory, and antifibrotic effects. The aim of this study was to evaluate the effect of topical pirfenidone added to conventional treatment in noninfected chronic diabetic foot ulcers. This was a randomized crossover study. Group 1 received topical pirfenidone plus conventional treatment for 8 weeks; after this period, they were switched to receive conventional treatment only for 8 more weeks. In group 2, the order of the treatments was the opposite. The end points were complete ulcer healing and size reduction. Final data were obtained from 35 ulcers in 24 patients. Fifty-two percent of ulcers treated with pirfenidone healed before 8 weeks versus 14.3% treated with conventional treatment only (*P* = 0.025). Between 8 and 16 weeks, 30.8% ulcers that received pirfenidone healed versus 0% with conventional treatment (*P* = 0.081). By week 8, the reduction in ulcer size was 100% [73–100] with pirfenidone versus 57.5% with conventional treatment [28.9–74] (*P* = 0.011). By week 16, the reduction was 93% [42.7–100] with pirfenidone and 21.8% [8–77.5] with conventional treatment (*P* = 0.050). The addition of topical pirfenidone to conventional treatment significantly improves the healing of chronic diabetic noninfected foot ulcers.

## 1. Introduction

Type 2 diabetes (T2D) is a chronic disease with an increasing incidence worldwide. The majority of T2D costs are derived from its complications. Diabetic foot is one of the most common and devastating complications of diabetes. It remains the leading cause of nontraumatic lower-extremity amputation [1, 2]. The annual incidence of diabetic foot ulcers varies between 1.9% and 2.2%, with a prevalence of 7.5% to 12% [3]. The risk of amputation is associated with the presence of sensory peripheral neuropathy, peripheral vascular disease, Charcot joint, ulceration, and the presence of infection [4]. Fifty-six percent of diabetic foot ulcers will develop infection and 20% of them will end up in a lower-extremity amputation [5].

A number of chemical mediators are involved in the tissue repair process such as cytokines and growth factors. Within this complex process, the transforming growth factor beta (TGF-*β*) plays a key role regulating the development, differentiation, growth, and apoptosis of most cells [6]. As inflammation develops, there is an increase in TGF-*β* production by inflammatory cells. TGF-*β* in turn activates monocytes by increasing gene expression of proinflammatory cytokines such as interleukin 1 (IL-1) and tumor necrosis factor alpha (TNF-*α*). Metalloproteinases (MMPs) are also important in the repair process of damaged tissues. The natural substrates of MMPs include major proteins of the extracellular matrix (ECM) such as collagen, elastin, and proteoglycans [7].

There have been advances in the management of diabetic ulcers. Therapies including growth factors, bioengineered skin, tissue grafts, hyperbaric oxygen, negative pressure wound therapy, and other novel approaches to stimulating wound healing have demonstrated healing rates of around 40% in noninfected diabetic foot ulcers [8].

1-Phenyl-5-methyl-2-[1H]-pyridone (pirfenidone) is a synthetic chemical molecule that acts as a selective cytokine regulator, providing its action by anti-inflammatory and specific antifibrotic properties. Pirfenidone acts as a modulator of TNF-*α*, TGF-*β*, fibroblast growth factor (FGF), platelet derived growth factor (PDGF), and vascular endothelial growth factor (VEGF), which are cytokines involved in the inflammatory-fibrotic process. This results in a reduced expression of TGF-*β* by a direct inhibition of furin, a proprotein convertase. These actions balance the production of MMPs [9]. These effects are associated with an improvement in reepithelialization, inflammation, and fibrosis.

Pirfenidone has shown utility in the treatment of patients with wounds, burns, and scars without serious adverse events [10]. Topical pirfenidone may improve healing of diabetic foot ulcers and could be an option as an adjuvant therapy. Therefore, the aim of this study is to evaluate the effect of pirfenidone added to conventional treatment on noninfected diabetic foot ulcers assessing the rate of complete wound closure and the change in ulcer size. In addition, safety of pirfenidone will be evaluated.

## 2. Material and Methods

### 2.1. Trial Design

This was a prospective controlled randomized crossover study. The protocol was approved by the Comite de Etica en Investigacion del Instituto Nacional de Ciencias Medicas y Nutricion. All subjects agreed to participate and provided informed consent before starting the study. The trial was registered under Clinical Trials NCT02222376.

### 2.2. Participants

Subjects who attended the Diabetic Foot Clinic at the Instituto Nacional de Ciencias Medicas y Nutricion Salvador Zubiran in Mexico City were recruited. Wagner grading system was used to classify the ulcers for inclusion. Grade 0 is a patient at risk or with a postulcerative diabetic foot without ulcer. Grade 1 is a full-thickness ulcer not involving tendon, capsule, or bone. Grade 2 is an ulcer that involves tendon or capsule, without abscess or osteomyelitis. Grade 3 is a deep ulcer with abscess or osteomyelitis. Grade 4 is an ulcer with gangrene in a portion of the forefoot. Finally, grade 5 is an ulcer with extensive gangrene [12].

The inclusion criteria were the following: men and women with T2D, being older than 18 years of age, being with a diabetic foot ulcer grade Wagner 1 or 2, ulcer size ≥1 cm^2^, and being with duration of at least 8 weeks. The exclusion criteria included infected ulcers, presence of an ankle brachial index (ABI) <0.4, ulcers due to a different cause such as venous insufficiency, inability to attend to the weekly evaluations, use of systemic or topical diabetic foot ulcer treatments, immunosuppressant treatment, connective tissue diseases, pregnancy, and lactation. None of the patients received antibiotic treatment previously or during the study. The elimination criteria were an attendance to <75% of the evaluations, allergy to pirfenidone, development of ulcer infection, and occurrence of other serious diseases requiring hospitalization.

### 2.3. Interventions

Conventional treatment consisted of weekly ulcer cleansing with saline, debridement using a surgical blade, maintenance of a moist environment, and covering with sterile gauzes. In addition, patients were instructed to perform daily cleansing with saline-moistened gauzes and offloading the affected extremity. Topical pirfenidone treatment consisted of applying pirfenidone over the ulcer twice a day.

All patients completed a pretreatment phase of 7 days receiving conventional treatment. After this week, participants were randomly assigned to one of the two groups. Group 1 received conventional treatment in combination with topical pirfenidone for the first eight weeks and at the end of this period they were switched to conventional treatment only for the remaining 8 weeks. Group 2 received conventional treatment only for the first 8 weeks and at the end of this period they were switched to conventional treatment in combination with topical pirfenidone for another 8 weeks. Patients were evaluated weekly during the 17 weeks at the diabetic foot clinic.

Weight, height, and blood pressure were measured using standardized techniques. The body mass index was calculated as the weight in kilograms divided by the squared height in meters. In addition, all ulcers were categorized using the classification of the International Working Group on the Diabetic Foot (IWGDF), abbreviated with the acronym PEDIS, which stands for perfusion, extent (size), depth (tissue loss), infection, and sensation (neuropathy) [13]. To evaluate the presence of lower-extremity vascular disease, the ABI was calculated and classified as follows: normal from 0.9 to 1.3, peripheral artery disease (PAD) ≤ 0.9, and severe PAD < 0.4. When the ABI was > 1.3, it was considered as a noncompressible vessel [11]. After debridement, the length and width of the ulcer were measured with a standard ruler, the maximal size was calculated in cm^2^, and a photograph was taken.

Biochemical parameters included complete blood cell count, erythrocyte sedimentation rate, C-reactive protein, glucose, creatinine, uric acid, total cholesterol, high density lipoprotein- (HDL-) cholesterol, low density lipoprotein- (LDL-) cholesterol, triglycerides, glycosylated hemoglobin, aspartate aminotransferase, alanine aminotransferase, and 24 h albuminuria.

Evaluation of metabolic control and adjustment of treatment were done as needed. Patients were reinforced about offloading their affected extremities, and adherence to treatment was assessed by requesting the empty tubes. Finally, possible harm including burning, redness, itching, and hypergranulation was monitored by physical examination and questioning. Unexpected adverse events were also recorded. Attribution to treatment was decided by an unblinded investigator.

### 2.4. Outcomes

Complete wound closure was defined as full epithelialization of the ulcer with absence of drainage. Percentage of closure was estimated by calculating the ulcer size in cm^2^ at visit 1 and at visit 8. The size at the end of each period was subtracted to the initial ulcer size (week 1 and week 8) and the change was estimated and expressed as a percentage.

### 2.5. Sample Size

A sample size of 50 ulcers was calculated estimating a 25% change in ulcer size with pirfenidone treatment, with an alpha error of 5% (*Z*_*α*_ = 1.96) and a beta error of 10% (power of 90%).

### 2.6. Randomization

Participants were randomly assigned in blocks to one of the two groups with the use of sealed envelopes using a random sequence numbers generator.

### 2.7. Statistical Analysis

The distribution of quantitative variables was analyzed with the Shapiro-Wilk test. Normal distributed variables were described as means and standard deviation. In the case of nonnormal distributed variables, median and interquartile ranges are used. Categorical variables were described as percentages and proportions. Differences between groups were evaluated with independent Student's *t*-tests or *U* Mann-Whitney tests, as appropriate. For categorical variables, chi-squared test was used. Statistical significance was considered with a two-sided *P* value <0.05. The statistical program SPSS version 20 was used to perform the analysis.

## 3. Results

### 3.1. Participant Flow

**Figure 1:**
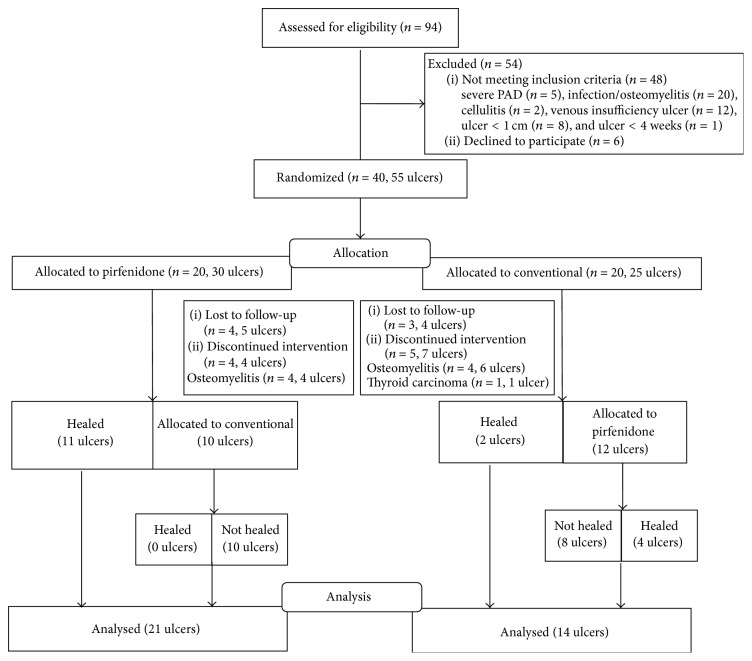
Flow diagram of patients during the study.

Forty patients with 55 ulcers were randomized to pirfenidone (*n* = 20) or conventional (*n* = 20) treatment. The flow diagram of randomized patients is shown in Figure 1.

### 3.2. Baseline Data

Participants' characteristics were not different between the groups except for the body mass index, which was slightly higher in the group assigned to receive conventional treatment first (29 vs 25.3 kg/m^2^, *P* = 0.048). We do not consider that this slight difference in the body mass index may have affected the results. These data are presented in Table 1.

Baseline ulcer size and depth were not different between groups. However, according to the ABI classification, in group 1 there were more patients with PAD and in group 2 there were more patients with noncompressible arteries. We excluded patients with severe PAD and do not consider that these differences affected the results.  Table 2 shows the baseline ulcer characteristics.

### 3.3. Outcomes

In the first eight weeks, 52.4% of the ulcers assigned to group 1 healed compared with 14.3% of group 2 (*P* = 0.025). After the crossover, the remaining 22 ulcers switched treatments and 30.8% of ulcers that received pirfenidone (during 8 to 16 weeks) healed compared with none in the conventional only group (*P* = 0.081). These figures are presented in Table 3.

**Figure 2:**
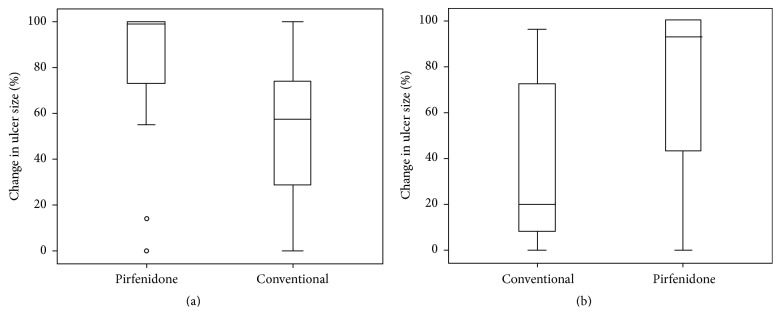
(a) Change in ulcer size expressed as percentage from baseline to 8 weeks. (b) Change in ulcer size expressed as percentage from 8 weeks to 16 weeks.

At 8 weeks, the median percentage reduction in ulcer size was 100% [73–100] in group 1 compared with a 57.5% [28.9–74] reduction in group 2, *P* = 0.011 (Figure 2(a)). At 16 weeks, the median percentage reduction was 93% [42.7–100] in the pirfenidone group compared with a 21.8% [16–77.5] size reduction in the conventional group, *P* = 0.050 (Figure 2(b)).

**Figure 3:**
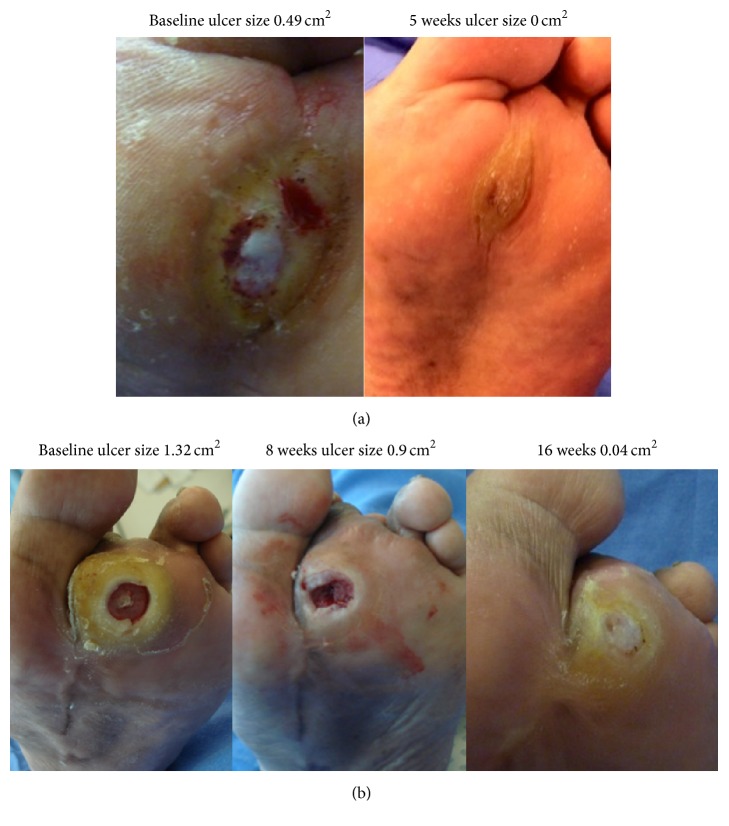
(a) Ulcer assigned to pirfenidone plus conventional treatment group during the first 8 weeks, that healed before crossover. (b) Ulcer assigned to conventional treatment only during the first 8 weeks, and crossover to pirfenidone plus conventional treatment.

Figure 3(a) shows an ulcer assigned to group 1 and Figure 3(b) an ulcer assigned to group 2.

### 3.4. Safety and Tolerability

No serious adverse events were observed during the course of the study in any treatment group. One patient in group 2 developed hypergranulation during pirfenidone treatment which resolved spontaneously. In group 2, a patient was diagnosed with thyroid carcinoma during conventional treatment and was eliminated from the study (Figure 1 and Table 4).

During the first 8 weeks, 10 ulcers (8 patients) developed osteomyelitis. Four ulcers (4 patients) were in group 1 and 6 ulcers (4 patients) in group 2; one of them had a supracondylar amputation. These patients were eliminated from the study (Table 4).

## 4. Discussion

This study demonstrates that pirfenidone added to conventional treatment is superior to conventional treatment alone. Treatment with pirfenidone decreased the size and increased the rate of complete healing of chronic noninfected diabetic foot ulcers. A greater number of ulcers achieved complete wound closure when receiving pirfenidone treatment.

Pirfenidone enhanced successful healing in addition to the standard ulcer care. A key component of the intervention was the weekly ulcer debridement and offloading the affected foot. Debridement enables removal of devitalized and necrotic tissue and promotes the beginning of the healing process [14]. The addition of pirfenidone accelerated the wound healing process.

Chronic diabetic foot ulcers represent a therapeutic challenge and their treatment involves debridement, frequent assessment, identification and treatment of infection, revascularization if indicated, and satisfactory offloading the foot [15].

Analysis of the evidence regarding the effectiveness of interventions to enhance healing of chronic diabetic foot ulcers is difficult. There are few controlled studies and the majority have methodological problems. There is not strong evidence to choose a specific dressing or topical medication in preference to another. Products designed to improve wound biochemistry and cell biology to promote wound healing demonstrate an ulcer healing rate between 40% and 80%. However, the evidence to support their use is not robust and further rigorously designed blinded trials are needed [8].

The results of this study are not generalizable due to the strict selection criteria. We excluded and eliminated patients with critical arterial insufficiency and infection to avoid confounders. Also, these conditions require specific and individualized treatment [16, 17]. A crossover design was chosen because otherwise it would not have been possible to control variables that could influence significantly the healing process such as ulcer size, ulcer depth, glycemic control, physical activity, and weight.

## 5. Conclusion

In summary, this study demonstrates that the addition of topical pirfenidone to conventional treatment significantly improves the healing of chronic diabetic noninfected foot ulcers.

## Figures and Tables

**Table 1:** Baseline characteristics of the studied population.

Variable	Group 1 (*n* = 20)	Group 2 (*n* = 20)	*P*
Male, number (%)	13 (65)	15 (75)	0.366
Age (years)	55.9 ± 14.2	54.7 ± 11.2	0.769
BMI, kg/m^2^	25.3 [23.3–30.7]	29.0 [25.8–33.4]	0.048
Systolic blood pressure, mmHg	130 [115–140]	130 [120–140]	0.859
Diastolic blood pressure, mmHg	70 [70–80]	70 [70–85]	0.318
Time from DM diagnosis, years	18.3 [14.3–28.0]	15.3 [9.6–21.0]	0.107
Glucose, mg/dL	148 [110–196]	136 [108–191]	0.988
Creatinine, mg/dL	1.2 [.99–2]	0.95 [.86–1.3]	0.059
Albuminuria, mg/24 h	277.4 [27.4–758]	87.4 [11–739.6]	0.241
A1c, %	8.2 [7.2–8.4]	8.6 [7.1–9.5]	0.184
Triglycerides, mg/dL	135.5 [109–192]	133.5 [97.5–251]	0.930
Total cholesterol, mg/dL	172 [137–178]	159 [137–183]	0.988
HDL cholesterol, mg/dL	44.2 ± 13.5	41.8 ± 8.8	0.503
LDL cholesterol, mg/dL	93.9 ± 33.2	90 ± 34.1	0.733
ALT, U/L	17 [13–23]	18 [13–30]	0.837
AST, U/L	18 [17–24]	19.5 [14–22]	0.937
Uric acid, mg/dL	6.9 ± 1.5	6.5 ± 1.7	0.480
Hemoglobin, g/dL	12.7 [11.7–13.5]	13.5 [12.2–15.4]	0.349
Hematocrit, %	38 [36–41.5]	39.9 [36.7–45.9]	0.388
Platelets, K/*μ*L	229.2 ± 54	239.2 ± 61.9	0.608
White blood cells, ×10^3^	7 ± 1.3	7.1 ± 1.2	0.868
ESR, mm/h	14 [5–34.5]	12 [6–32]	0.987
C-reactive protein, mg/dL	.28 [.18–.94]	.35 [.18–.93]	0.690

Data is expressed as mean ± SD or median [interquartile range]. BMI: body mass index calculated as weight in kilograms divided by the square of height in meters; DM: diabetes mellitus; A1c: glycated hemoglobin; HDL: high density lipoprotein; LDL: low density lipoprotein; ALT: alanine aminotransferase; AST: aspartate aminotransferase; ESR: erythrocyte sedimentation rate.

**Table 2:** Baseline ulcer characteristics.

Characteristics	Total (*n* = 35)	Group 1 (*n* = 21)	Group 2 (*n* = 14)	*P*
Size, cm^2^	1.32 [0.49–6.55]	0.75 [0.40–7.56]	1.40 [1.08–3.41]	0.630

Depth				0.955
Superficial	15 (42.9)	9 (42.9)	6 (42.9)	
Dermis, muscle, tendon	18 (51.4)	11 (52.4)	7 (50.0)	
All layers	2 (5.7)	1 (4.8)	1 (7.1)	

ABI right				0.406
Normal	7 (20)	5 (23.8)	2 (14.3)	
Noncompressible	28 (80)	16 (76.2)	12 (85.7)	

ABI left^*∗*^				0.003
Normal	11 (32.4)	10 (50)	1 (7.1)	
PAD	3 (8.8)	3 (15)	0	
Noncompressible	20 (58.8)	8 (35)	13 (92.9)	

Depth				0.970
Superficial	15 (41.6)	9 (40.9)	6 (42.9)	
Dermis, muscle, tendon	18 (50)	11 (50)	7 (50)	
All layers	3 (8.3)	2 (9.1)	1 (7.1)	

Data expressed in median [interquartile range] or number (percentage).

ABI: ankle brachial index; PAD: peripheral arterial disease.

ABI was classified as follows: normal from >0.9–1.3, ≤0.9 PAD, <0.4 severe PAD, and >1.3 noncompressible vessel [11].

^*∗*^In one patient left ABI could not be estimated due to history of amputation.

**Table 3:** Complete healing in the treatment groups.

	All ulcers *N* (%)	Pirfenidone *N* (%)	Conventional *N* (%)	*P*
Ulcer healing < 8 weeks (35 ulcers at the beginning)	13 (37.1)	11 (52.4)	2 (14.3)	0.025

Ulcer healing 8–16 weeks (22 ulcers at the beginning)	4 (17.4)	4 (30.8)	0	0.081

**Table 4:** Adverse events.

	Group 1		Group 2	
	Weeks 0 to 7	Weeks 8 to 16	Weeks 0 to 7	Weeks 8 to 16
	Pirfenidone	Conventional treatment	Conventional treatment	Pirfenidone
Osteomyelitis	4 (eliminated)	0	6 (eliminated)	0
Hypergranulation	0	0	0	1
Thyroid carcinoma	0	0	1 (eliminated)	0

Total AE	4	0	7	1

## References

[1] Van Houtum W. H., Lavery L. A. (1996). Outcomes associated with diabetes-related amputations in the Netherlands and in the state of California, USA. *Journal of Internal Medicine*.

[2] van Houtum W. H., Lavery L. A., Harkless L. B. (1996). The impact of diabetes-related lower-extremity amputations in the Netherlands. *Journal of Diabetes and Its Complications*.

[3] Lavery L. A., Armstrong D. G., Wunderlich R. P., Tredwell J., Boulton A. J. M. (2003). Diabetic foot syndrome: evaluating the prevalence and incidence of foot pathology in Mexican Americans and non-Hispanic whites from a diabetes disease management cohort. *Diabetes Care*.

[4] Boulton A. J. (1990). Lawrence lecture. The diabetic foot: neuropathic in aetiology?. *Diabetic Medicine*.

[5] Wu S. C., Driver V. R., Wrobel J. S., Armstrong D. G. (2007). Foot ulcers in the diabetic patient, prevention and treatment. *Vascular Health and Risk Management*.

[6] Teller P., White T. K. (2009). The physiology of wound healing: injury through maturation. *Surgical Clinics of North America*.

[7] Wahl S. M. (1992). Transforming growth factor beta (TGF-*β*) in inflammation: a cause and a cure. *Journal of Clinical Immunology*.

[8] Game F. L., Apelqvist J., Attinger C. (2016). Effectiveness of interventions to enhance healing of chronic ulcers of the foot in diabetes: a systematic review. *Diabetes/Metabolism Research and Reviews*.

[9] Schaefer C. J., Ruhrmund D. W., Pan L., Seiwert S. D., Kossen K. (2011). Antifibrotic activities of pirfenidone in animal models. *European Respiratory Review*.

[10] Macias-Barragan J., Sandoval-Rodriguez A., Navarro-Partida J., Armendariz-Borunda J. (2010). The multifaceted role of pirfenidone and its novel targets. *Fibrogenesis Tissue Repair*.

[11] Olin J. W., Kaufman J. A., Bluemke D. A. (2004). Atherosclerotic vascular disease conference: writing group IV: imaging. *Circulation*.

[12] Wagner F. W. (1987). The diabetic foot. *Orthopedics*.

[13] Schaper N. C. (2004). Diabetic foot ulcer classification system for research purposes: a progress report on criteria for including patients in research studies. *Diabetes/Metabolism Research and Reviews*.

[14] Saxena V., Hwang C.-W., Huang S., Eichbaum Q., Ingber D., Orgill D. P. (2004). Vacuum-assisted closure: microdeformations of wounds and cell proliferation. *Plastic and Reconstructive Surgery*.

[15] Hopf H. W., Humphrey L. M., Puzziferri N., West J. M., Attinger C. E., Hunt T. K. (2001). Adjuncts to preparing wounds for closure: hyperbaric oxygen, growth factors, skin substitutes, negative pressure wound therapy (vacuum-assisted closure). *Foot and Ankle Clinics*.

[16] Lipsky B. A., Berendt A. R., Cornia P. B. (2012). 2012 infectious diseases society of America clinical practice guideline for the diagnosis and treatment of diabetic foot infections. *Clinical Infectious Diseases*.

[17] Weitz J. I., Byrne J., Clagett G. P. (1996). Diagnosis and treatment of chronic arterial insufficiency of the lower extremities: a critical review. *Circulation*.

